# Placental Location Site and Associated Intrapartum, Postpartum, and Neonatal Complications: A Comprehensive Review and Meta-Analysis

**DOI:** 10.3390/jcm14051649

**Published:** 2025-02-28

**Authors:** Dayna D. Whitcombe, Zhuopei Hu, Songthip T. Ounpraseuth, Everett F. Magann

**Affiliations:** 1 Department of Obstetrics and Gynecology, University of Arkansas for Medical Sciences (UAMS), 4301 W. Markham Street, Slot # 518, Little Rock, AR 72205-7199, USA; 2 Departments of Biostatistics, University of Arkansas for Medical Sciences (UAMS), Little Rock, AR 72205-7199, USA

**Keywords:** placental location, intrapartum complications, postpartum complications, neonatal complications

## Abstract

**Background/Objectives:** Adverse intrapartum, postpartum, and neonatal complications have been linked to placenta implantations sites. However, different reviews have led to contrasting conclusions about placental locations and pregnancy outcomes. We aim to determine if there is a relationship between the placental implantation site and intrapartum, postpartum, and neonatal outcomes. **Methods:** The Meta-analysis of Observational Studies in Epidemiology (MOOSE) guidelines were followed during this review. The literature search used PubMed, CINAHL, and Embase. Years searched was not a study limitation. Only articles in English were included. Two authors reviewed the abstracts. **Results:** Of 40 articles identified as specific to intrapartum, postpartum, and/or neonatal complications (with some articles overlapping categories), 19 included intrapartum complications, 19 included postpartum complications, and 22 included neonatal complications. Pregnancies with a midline placenta (compared to a lateral placenta) had a greater likelihood of macrosomia/LGA infant (odds ratio (OR), 1.52 (95% CI: 1.22–1.90)) and lesser likelihood to have non-cephalic presentation (OR, 0.17 (95% CI: 0.06–0.51)), FGR/SGA infant (OR, 0.68 (CI: 0.55–0.85)), and retained placenta (OR, 0.33 (95% CI: 0.22–0.50)). Pregnancies with a low-lying placenta (compared to within uterine corpus) were more likely to have non-elective cesarean section (OR, 1.94 (95% CI: 1.06–3.55)) and postpartum hemorrhage (OR 1.49 (95% CI: 1.12–1.97)). **Conclusions:** Significant associations between placental location site and intrapartum complications (non-cephalic presentation, non-elective cesarean section), postpartum complications (postpartum hemorrhage, retained placenta), and neonatal complications (FGR/SGA, Macrosomia/LGA) were identified. There were no significant associations identified between the placental location site and several neonatal complications, including Apgar < 7 at 5 min, NICU admission, IUFD, and neonatal death.

## 1. Introduction

The fetal anatomic survey, which includes an assessment of the uterus, adnexa, fetal anatomy, amniotic fluid volume, and placental implantation site, is extensively utilized in pregnancy. Placental location permits researchers to establish if specific placental location sites are associated with adverse pregnancy outcomes.

Adverse intrapartum, postpartum, and neonatal outcomes have been linked to placental locations; however, different investigations have led investigators to conflicting assumptions. Small numbers and differing gestational ages when studies were performed have led to differing findings. The lack of a common classification system to categorize the placenta site of implantation makes it difficult to assess existing studies.

The study was undertaken to determine if there is an association between the placental location and intrapartum, postpartum, and neonatal pregnancy outcomes. The study was conducted using a comprehensive literature search by a university research librarian with subsequent systematic review and meta-analysis.

## 2. Objectives

### 2.1. Methods

This prospective systematic review was registered with PROSPERO [PROSPERO 2022: CRD42022289290] and conducted following the Meta-analysis of Observational Studies in Epidemiology (MOOSE) guidelines (see Table S1) [1]. This is a follow-up study of a prior study that evaluated placental locations and adverse antepartum pregnancy complications [2]. The initial search for the first study was undertaken on 1 February 2018. At that time, we found 40 studies that included not only antepartum complications but also postpartum and neonatal outcomes [2]. We then decided to look at the intrapartum, postpartum, and neonatal outcomes related to placental location in a second paper. To make sure that we had not missed any studies that might have been published since the original search, we performed a second literature search using the same search terms that covered 1 January 2018–31 December 2022. The second search used the same search engines PubMed, CINAHL, and Embase and the same search terms “placental location” OR “placenta location” AND “pregnancy outcome(s)” OR “pregnancy complications” [2]. Limitations were not put on the years searched. The quality of evidence was determined using the Newcastle–Ottawa Scale (NOS) (see Table S2) [3]. Meta-analysis of published data was verified by our IRB to not require informed consent.

Forty studies were identified with varied definitions of placental implantation sites among the studies. As we did in the initial study, to maintain a common language between studies, descriptions of placental location categories for each study were analyzed and then divided into five main groups (the grouping details of placental locations are detailed in our first paper on placental locations [2]). Viewing the uterus, both transverse and sagittal, placental locations were categorized as central, fundal, lateral, uterine corpus, and LUS. LUS was defined as a placental location in the uterus below the uterine corpus. Transvaginal vs. transabdominal sonography was used selectively in the 16 studies with placentas classified as low-lying or previa. There were six studies that did not specify in the materials and methods which type of ultrasound scanning was used; three of the studies used both techniques, three studies used only transabdominal sonography, two used only transvaginal sonography, one used manual exploration following delivery, and one used “placentography” (radiographic) technique [2] Details of each study, including the placental location in the original study and the classification of the placental location used for this study, are shown in Table 1 [3,4,5,6,7,8,9,10,11,12,13,14,15,16,17,18,19,20,21,22,23,24,25,26,27,28,29,30,31,32,33,34,35,36,37,38,39,40,41,42,43].

Placental location was associated with intrapartum, postpartum, and neonatal outcomes. The detection process of the placental locations is described in the Section 2.1 of the study. Every identified study, including those from the time period when ultrasound was not commonly used, was included. Two studies identified the placental locations after fetal delivery.

The intrapartum, postpartum, and neonatal complications assessed were identified by our investigation of the literature. Intrapartum complications included were non-cephalic presentation and non-elective cesarean section. The postpartum complications evaluated were postpartum hemorrhage (PPH) and retained placenta. The fetal/neonatal complications evaluated were fetal growth restriction (FGR)/small for gestational age (SGA), macrosomia/ large for gestational age (LGA), 5 min APGAR score < 7, NICU admission, intrauterine fetal demise (IUFD), and neonatal death.

### 2.2. Statistical Analysis

All statistical analyses were performed at a two-sided significant level of 0.05 and analyses were carried out using the R package ‘metafor’ and ‘meta’ (R Foundation for Statistical Computing, Vienna, Austria) [44,45]. Additionally, all analyses were performed in accordance with the Cochrane Handbook for Systematic Reviews of Interventions guidelines [46]. For each intrapartum, postpartum, and neonatal complication of interest and various categories of placental location comparisons, respectively, the calculated effect estimates were pooled to obtain the overall effect expressed as odds ratio (OR) along with the corresponding 95% confidence interval (CI).

We also estimated the pooled proportions with 95% CI of each placental location comparison (e.g., midline vs. lateral) using the R package ‘metapropr [45]. We examined the magnitude of between-study heterogeneity after correcting for statistical artifacts using the Q-statistic and Higgins’ I^2^ statistic with levels < 40%, between 30% and 60%, between 50% and 90%, and >75% equating to low, moderate, substantial, and high levels of heterogeneity [46]. We note that when the number of studies is small, the Q-statistic is typically underpowered for detecting true heterogeneity; thus, our results are based on pooled data using a random effects model. We also examine inter-study variance given by ꞇ^2^, which provides the estimated standard deviation of the underlying effects across the studies. For neonatal complications of interest with at least 6–8 studies available to perform a meta-analysis, we used funnel plots and Egger’s asymmetrical test [47] to assess the potential existence of small study bias. If potential publication bias was detected, we adjusted our results using a nonparametric trim-and-fill approach [48]. Finally, a sensitivity analysis was carried out using a leave-one-out method to evaluate the robustness of our results.

## 3. Results

Our search of the literature recognized 203 abstracts. All abstracts were read. The entire article was obtained and read for any abstract associating placenta implantation site and pregnancy outcomes. There were 43 studies found. References of pertinent articles were examined and 31 additional articles were found.

**Figure 1:**
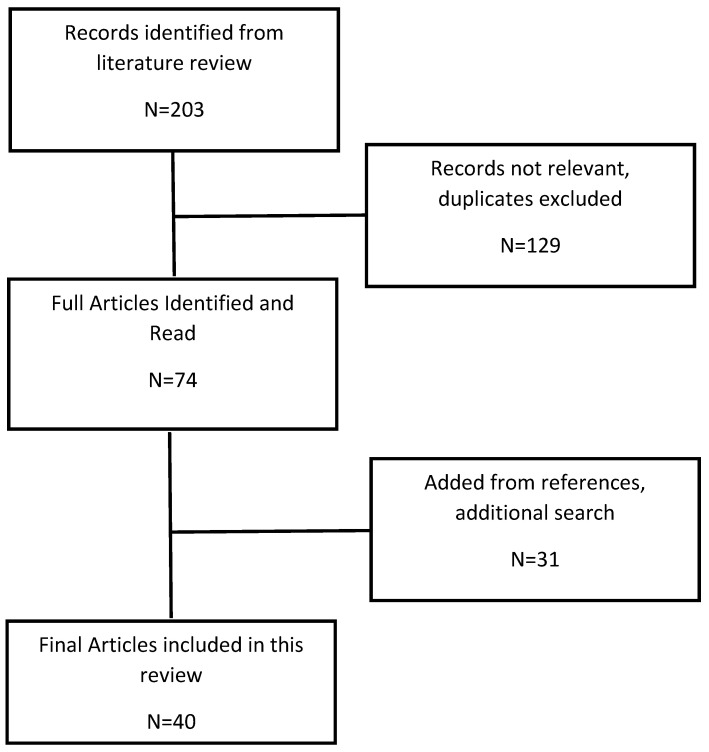
Literature Review Diagram.

From the 74 discovered studies, 40 articles specific to intrapartum, postpartum, and/or neonatal complications were recognized and are the basis of this systematic review and meta-analysis (Figure 1). A summary of the characteristics of the final 40 relevant studies, including outcomes evaluated in each study, can be found in Table 1. Our sensitivity analysis using the leave-one-out method did not indicate extreme influences of single studies.

### 3.1. Intrapartum Complications

1. Non-Cephalic Presentation

**Figure 2:**
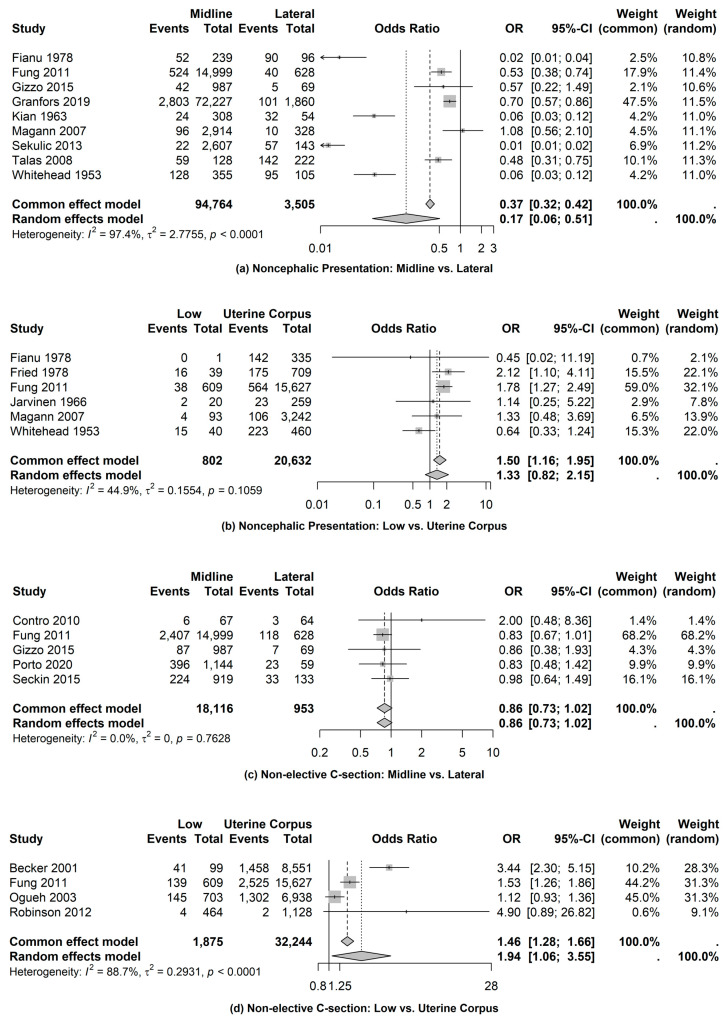
Intrapartum complications: non-cephalic presentation and non-elective cesarean section [6,10,13,14,15,16,18,21,23,29,31,34,35,37,38,40,42].

We identified nine studies that were used to compare the odds of identifying non-cephalic presentation based on examinations of placental location using midline versus lateral location. The combined data from the midline location were 94,764 observations compared to 3505 cases with placenta in the lateral location. The odds of identifying non-cephalic presentation for examinations with midline placental location are 0.17 times (95% CI: 0.06, 0.51) the odds of lateral placental location (see Figure 2). The pooled proportion of non-cephalic presentation based on midline location was 0.14 (95% CI: 0.06, 0.23) compared to 0.41 (95% CI: 0.17, 0.65) using lateral location. In follow-up, we examined the odds of identifying non-cephalic presentation based on examinations of low uterine segment versus uterine corpus. A total of six studies were utilized for this comparison (see Figure 2). The pooled proportion of non-cephalic presentation based on the low uterine segment was 0.18 (95% CI: 0.03, 0.32) compared to 0.20 (95% CI: 0.07, 0.32) using uterine corpus. While the odds of identifying non-cephalic presentation based on low uterine segment examinations were 33% higher compared to uterine corpus, they were not statistically significant (OR = 1.33; 95% CI: 0.82, 2.15).

2. Non-Elective Cesarean Section

We identified five studies that were used to compare the odds of non-elective cesarean section based on examinations of placental location using midline versus lateral location. The combined data from the midline location were 18,116 observations compared to 953 cases with lateral placental locations. The odds of non-elective cesarean section for examinations based on midline are 0.86 times (95% CI: 0.73, 1.02) the odds of lateral examinations (see Figure 2), which was not statistically significant. The pooled proportion of non-elective cesarean section based on midline location was 0.16 (95% CI: 0.08, 0.24) compared to 0.19 (95% CI: 0.08, 0.30) using lateral location. In follow-up, we examined the odds of non-elective cesarean section based on examinations of the low uterine segment versus uterine corpus. A total of four studies were utilized for this comparison (see Figure 2). The pooled proportion of non-elective cesarean section based on the low uterine segment was 0.21 (95% CI: 0.05, 0.37) compared to 0.14 (95% CI: 0.08, 0.21) using uterine corpus. The odds of non-elective cesarean section based on low uterine segment examinations were 94% higher compared to uterine corpus (OR = 1.94; 95% CI: 1.06, 3.55).

### 3.2. Postpartum Complications:

3. Postpartum Hemorrhage (PPH)

**Figure 3:**
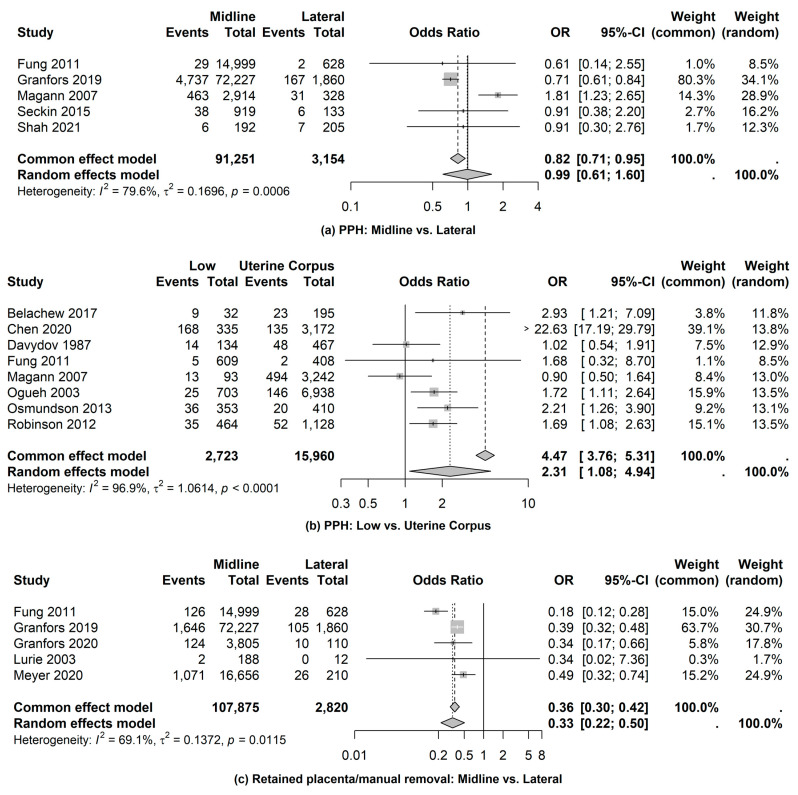
Postpartum complications: postpartum hemorrhage and retained placenta [7,9,11,15,17,18,28,29,30,31,33,35,37,39].

Based on the five studies used to compare the odds of postpartum hemorrhage, there was no statistical difference between examination of placental location using midline versus lateral location (OR = 0.99; 95% CI: 0.61, 1.60) (see Figure 3). However, examination of placental location based on the low uterine segment was statistically different compared to uterine corpus. More specifically, the odds of postpartum hemorrhage were 49% higher among the low uterine segment compared to the uterine corpus (OR = 1.49; 95% CI: 1.12, 1.97) (see Figure 3). The pooled proportion of postpartum hemorrhage in the low uterine segment group was 0.17 (95% CI: 0.08, 0.26) compared to 0.06 (95% CI: 0.03, 0.10) for uterine corpus.

4. Retained Placenta

For the retained placenta postpartum complication, there were sufficient studies to compare the odds of a retained placenta based only on examinations of placental location using midline versus lateral location. The pooled proportion of a retained placenta based on midline among the selected studies was 0.03 (95% CI: 0.01, 0.04) compared to 0.07 (95% CI: 0.04, 0.1) for lateral location. The combined data from the midline location were 107,875 observations compared to 2820 cases with lateral placenta location. The odds of a retained placenta for examinations based on midline are 0.33 times (95% CI: 0.22, 0.50) the odds of midline examinations (see Figure 3).

### 3.3. Neonatal Complications

5. FGR/SGA

**Figure 4:**
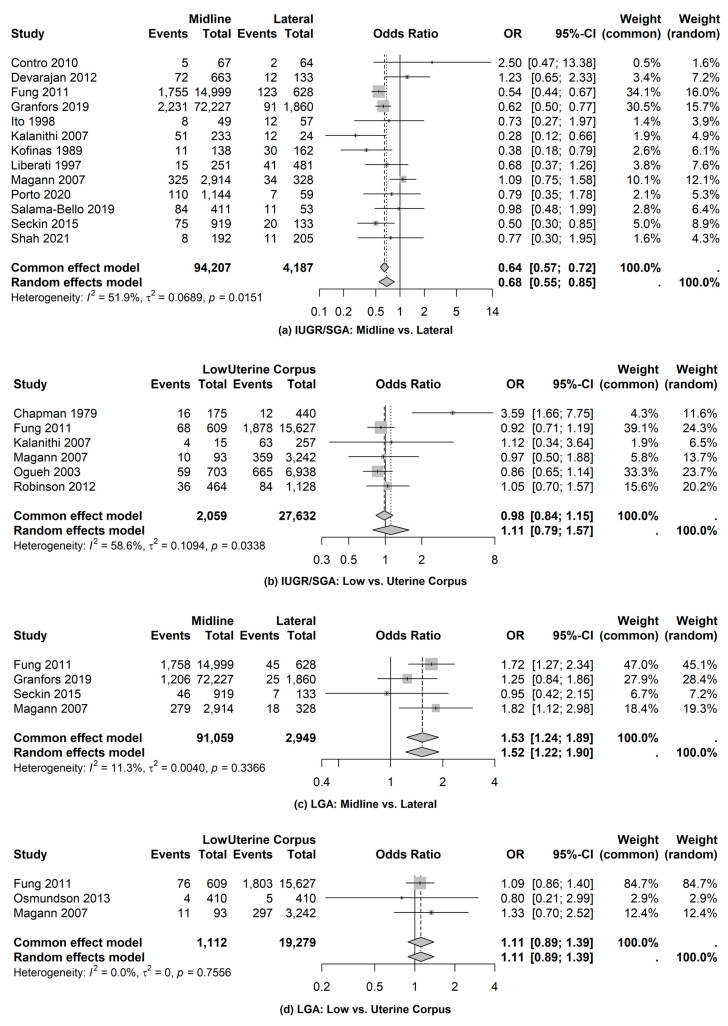
Fetal/neonatal complications: FGR/SGA and macrosomia/LGA [8,10,12,15,18,20,22,24,26,29,31,33,34,35,36,37,39].

We identified 13 studies that were used to compare the odds of FGR/SGA based on examinations of placental location using midline versus lateral location. The combined data from the midline location were 94,207 observations compared to 4187 cases with lateral placenta location. The odds of FGR/SGA for examinations based on midline are 0.68 times (95% CI: 0.55, 0.85) the odds of lateral examinations (see Figure 4). The pooled proportion of FGR/SGA based on a midline location was 0.10 (95% CI: 0.07, 0.12) compared to 0.13 (95% CI: 0.09, 0.17) using a lateral location. As a follow-up set of analyses, we examined the odds of FGR/SGA based on examinations of the low uterine segment versus uterine corpus. A total of six studies were utilized for this comparison (see Figure 4). The pooled proportion of FGR/SGA based on the low uterine segment was 0.09 (95% CI: 0.08, 0.11) compared to 0.10 (95% CI: 0.07, 0.12) using uterine corpus. While the odds of FGR/SGA based on low uterine segment examinations were 11% higher compared to uterine corpus, it was not statistically significantly different (OR = 1.11; 95% CI: 0.79, 1.57).

6. Macrosomia/LGA

Four studies were used to examine the odds of macrosomia based on examinations of placental location using midline versus lateral location. The combined data from the midline location were 91,059 observations compared to 2949 cases with a lateral placenta. The odds of macrosomia for examinations based on midline are 52% higher than the odds with the placenta in the lateral location (OR = 1.52; 95% CI: 1.22, 1.90) (see Figure 4). The pooled proportion of macrosomia on the midline location was 0.08 (95% CI: 0.04, 0.12) compared to 0.05 (95% CI: 0.02, 0.07). As a follow-up analysis, the odds of macrosomia were examined based on examinations of the low uterine segment versus uterine corpus. A total of three studies were utilized for this comparison (see Figure 4). Based on the comparison of low uterine segment examinations versus uterine corpus, the difference was not statistically significant (OR = 1.11; 95% CI: 0.89, 1.39).

7. Apgar < 7 at 5 Minutes

**Figure A1:**
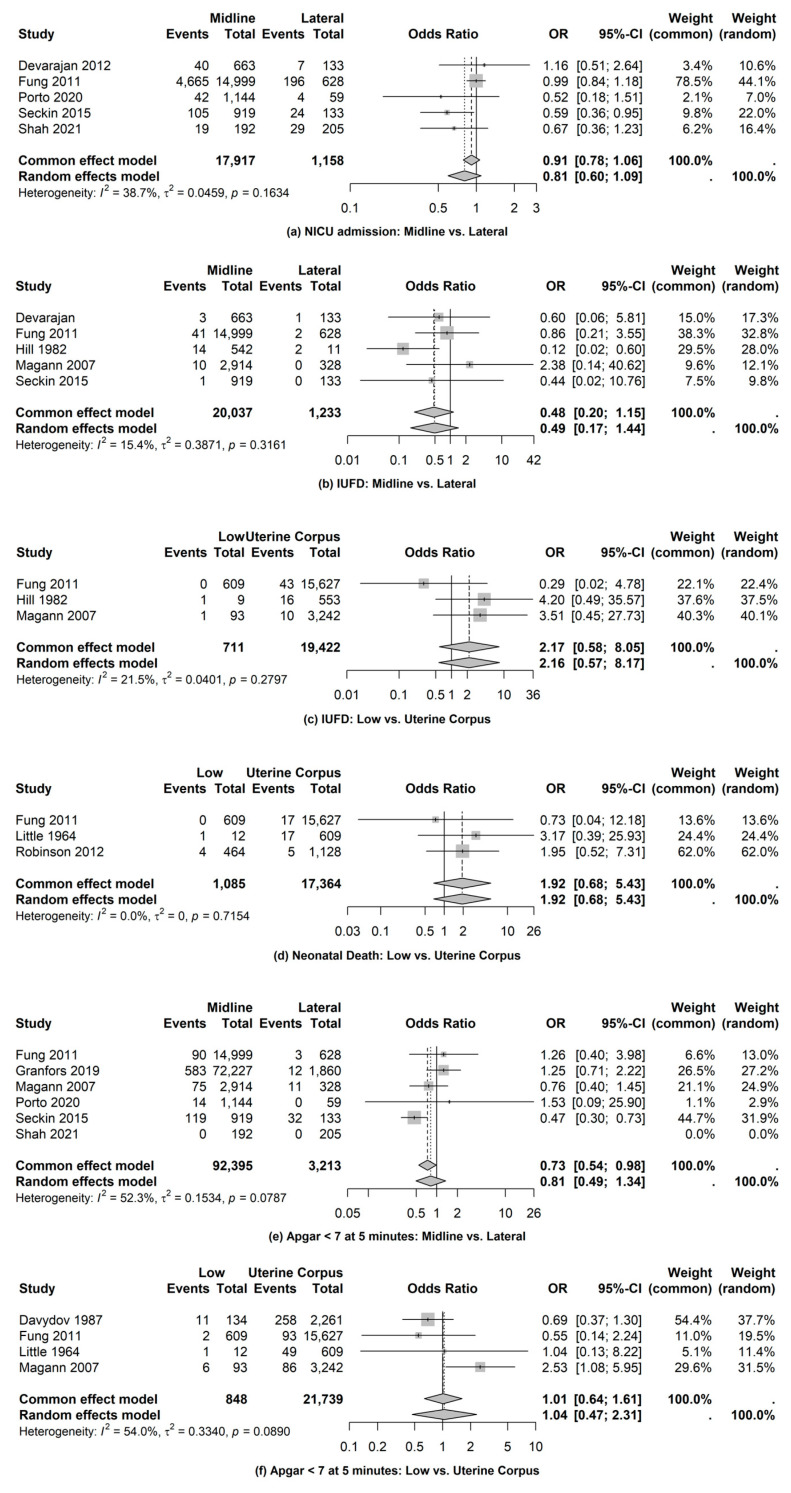
Intrapartum and postpartum perinatal complications [11,12,15,18,19,27,29,34,35,37,39].

We identified six studies used to compare the odds of an Apgar < 7 at 5 min based on examinations of placental location using midline versus lateral location. The combined data from the midline location were 92,395 observations compared to 3213 observations for the placenta in the lateral location. The odds of an Apgar < 7 at 5 min for examinations based on midline are 0.81 times (95% CI: 0.49, 1.34) the odds of lateral examinations (see Figure A1), which was not statistically significant. The pooled proportion of an Apgar < 7 at 5 min based on midline location was 0.04 (95% CI: 0.001, 0.08) compared to 0.04 (95% CI: 0.00, 0.11) using lateral location. As a follow-up, we examined the odds of an Apgar < 7 at 5 min based on examinations of the low uterine segment versus uterine corpus. A total of four studies were utilized for this comparison (see Figure A1). The pooled proportion of an Apgar < 7 at 5 min based on the low uterine segment was 0.05 (95% CI: 0.0, 0.10) compared to 0.05 (95% CI: 0.01, 0.09) using uterine corpus. There was no association between an Apgar < 7 at 5 min and placenta location at either the low uterine segment or uterine corpus (OR = 1.04; 95% CI: 0.47, 2.31).

8. NICU Admission

For NICU admissions of neonatal complications, there were sufficient studies to compare the odds of NICU admissions based only on examinations of placental location using midline versus lateral location. The pooled proportion of NICU admission based on midline among the selected studies was 0.13 (95% CI: 0.03, 0.22) compared to 0.15 (95% CI: 0.06, 0.25) for lateral location. The combined data based on five studies from the midline location were 17,917 observations compared to 1158 cases with a lateral placenta location. The odds of NICU admission for examinations based on midline are 0.81 times the odds of midline examinations (see Figure A1); however, it was not statistically significant (95% CI: 0.60,1.09).

9. IUFD

Based on five studies used to compare the odds of IUFD, there was no statistical difference between examination of placental location using midline versus lateral location (OR = 0.49; 95% CI: 0.17, 1.44) (see Figure A1). While the odds of IUFD were much higher based on lower uterine segment placental location compared to the uterine corpus, it was not statistically significant (OR = 2.16; 95% CI: 0.57, 8.17). The analysis was based on three studies, and the combined data from the lower uterine segment included 711 observations compared to 19,422 cases with placenta in the uterine corpus location (See Figure A1).

10. Neonatal Death

For neonatal death, the analysis was limited to three studies examining the odds of neonatal death based on placental location at the lower uterine segment versus uterine corpus. While the odds of neonatal death were higher based on placental location in the lower uterine segment compared to the uterine corpus, it was not statistically significant (OR = 1.92; 95% CI: 0.68, 5.43) (see Figure A1).

## 4. Discussion

### 4.1. Principal Findings

Placental location does influence some pregnancy outcomes. There are significant links between the placental implantation site and intrapartum complications (non-cephalic presentation, non-elective cesarean section), postpartum complications (postpartum hemorrhage, retained placenta), and neonatal complications (FGR/SGA, macrosomia/LGA).

Based on the intrapartum complications analyzed in this study, there was a significant association between non-cephalic presentation and placental location. The risk of a fetus with non-cephalic presentation was lower when the placenta was located midline compared to lateral. Additionally, and as would be expected, the risk of a non-elective cesarean section was statistically higher, with placentas located in the lower uterine segment compared to the uterine corpus.

There were two major findings in this study regarding placenta location and postpartum complications. First, the risk of postpartum hemorrhage (PPH) was significantly increased if the placenta was located within the lower uterine segment compared to the uterine corpus. This finding outcome is not unique as the relationship between placentas within the lower uterine segment, including marginal and complete placenta previa, tends to have a high elevated risk of bleeding following delivery. Secondly, the possibility of a retained placenta was significantly lower when the placenta was located midline compared to laterally. This finding is also reasonable as the lateral location in this study includes those placentas located at the cornua of the uterus, where a portion of placental tissue is more likely to be retained.

Out of the six neonatal complications evaluated in relation to placental location (FGR/SGA, macrosomia/LGA, Apgar < 7 at 5 min, NICU admission, IUFD, and neonatal death), there were two major findings. First, the risk of FGR/SGA was lower when placentas were located midline versus lateral. Additionally, and in congruence with the first finding, the risk of macrosomia/LGA was more likely when the placenta was located midline versus in the lateral position.

### 4.2. Strengths and Limitations

A strength of this study is the inclusion of relevant studies on placental location and its association with various intrapartum, postpartum, and neonatal complications based on two literature searches, one which covered 1 January 2000 to 31 December 2018 and the second which covered 1 January 2018–31 December 2022. The included studies had a low risk of bias overall, and many patients were included in the analysis. Moreover, the meta-analysis was registered through PROSPERO and was conducted according to MOOSE guidelines, utilized a comprehensive search strategy, and all studies underwent quality assessment.

Two of the main limitations of the study were how the placental locations were determined, the gestational age at which the placental location was determined, and how placental locations were grouped. The number of location sites identified in our study is a limitation because it is not possible, with the variance in the placental location site definitions between studies, to compare the studies without combining some placental sites to make comparisons. Despite this limitation, by combining the location sites, larger numbers within sites and pregnancy complications were able to be evaluated, and thus, added strength to our study. The question also arises that some studies evaluated placental locations at 16–18 weeks while other studies used third-trimester assessments. Could placental migration impact study findings? We consolidated placental locations for this study into five locations based on an earlier study that had characterized locations into nine groups in 2526 singleton pregnancies, of which 1336 were serially assessed five times during gestation (16–18, 24, 28, 34 weeks) [49]. The primary outcomes observed were that the posterior high implantation location was the most common and that posterior migration of the placenta was more likely to be seen than anterior migrations. No other significant migrations were observed. Therefore, although migration does occur, the influence of those migrations on outcomes, other than the low-lying placentas in relation to the cervix and their pregnancy outcomes, would be thought to be minimal. Another limitation of this study is that we did not do a sub-analysis of confounders for maternal morbidities, which was not performed in many of the included studies.

Additionally, although most studies utilized ultrasonography to identify the placental location, some of the older studies utilized different techniques, such as manual evaluation following delivery or “placentography” (radiographic technique). These studies were also included in our analysis. This was performed since our objective was not about how the placenta locations were agreed upon but the correlation of the locations (used at the time of the study) to pregnancy complications. The criteria for study inclusion were set prior to literature review and between-study heterogeneity was accounted for using a random-effects model to reduce study heterogenicity. One final limitation was the fact that all data are from observational studies (case-control, cohort) rather than randomized controlled studies.

### 4.3. Clinical Implications and Areas of Future Research

Many of the findings of this study further confirm current beliefs and can be used to further validate clinical practices. For instance, a low-lying placenta has a higher risk of postpartum hemorrhage than a placenta located within the uterine corpus. With this knowledge, a clinician and ancillary staff can be better prepared to manage a postpartum hemorrhage with easily accessible uterotonic medications and tamponade devices, for instance. Based on this study, there is a higher likelihood of having a retained placenta with laterally located placentas. In instances of persistent postpartum bleeding outside what is generally considered normal or in cases of postpartum fever, a high level of suspicion should be maintained in the setting of lateral/cornual placentas and a bedside ultrasound can be utilized for further evaluation. Due to the association of laterally located placentas and FGR, an additional or third-trimester ultrasound may be undertaken to evaluate fetal growth.

A potential opportunity for future research related to placental location and its impact on pregnancy complications would be to undertake a large prospective multi-center study with similar placental location definitions in the second trimester with adequate follow-up or pregnancy outcomes. Additional sub-analyses could also be undertaken, such as further dividing midline placentation into anterior, posterior, and fundal to evaluate pregnancy-related complications.

## 5. Conclusions

Placental location does influence some complications of pregnancy. Significant associations between the placental location site and the following were identified: intrapartum complications (non-cephalic presentation, non-elective cesarean section), postpartum complications (postpartum hemorrhage, retained placenta), and neonatal complications (FGR/SGA, macrosomia/LGA). There were no significant associations identified between the placental location site and several neonatal complications, including Apgar < 7 at 5 min, NICU admission, IUFD, and neonatal death.

## Figures and Tables

**Table 1:** Details of Each Study.

Reference	Year Published	Study Type	Gestational Age (GA)	Number	Original Placental Location	Placental Location in this Study	Outcomes
Baba [4]	2014	Retrospective Case Control	Time of delivery	205	Previa	Previa	PPH
Bardin [5]	2022	Retrospective Cohort	Within 3 days before delivery	5425	Anterior	Anterior	SGA
					Posterior	Posterior	Macrosomia
					Fundal	Fundal	
					Other	--	
Becker [6]	2001	Prospective Cohort	20–23 weeks	8650	Normal	High (Above LUS)	Cesarean Section
					Low	Low	
					Overlapping	Previa	
Belachew [7]	2017	Prospective Cohort	28–30 weeks	400	Anterior	Central	PPH
					Posterior/Fundal	Central/Fundal	Retained Placenta
					Low Anterior	Low Lying	
					Low Posterior	Low Lying	
					Anterior Previa	Previa	
					Posterior Previa	Previa	
Chapman [8]	1979	Prospective Cohort	16–24	615	Low	Low	SGA
					Other	High (Above LUS)	
Chen [9]	2020	Retrospective Cohort	Within 1 Week of Delivery	3722	Anterior	Central	PPH
					Posterior	Central	
					Lateral or Fundal	High (Above LUS)	
Contro [10]	2010	Prospective Cohort	20–22 weeks	131	Central	Central	Non-Elective Cesarean Section
					Lateral	Lateral	SGA
Davydov [11]	1987	Retrospective Cohort	Time of delivery	2396	Fundal	Fundal	PPH
					Uterine Body	Central	APGAR < 7
					Lower Segment	Low	Non-Elective Cesarean Section
Devarajan [12]	2012	Retrospective Cohort	16–24 weeks	796	Lateral	Lateral	SGA
					Central/Fundal	Central/Fundal	NICU Admission
Fianu [13]	1978	Prospective Cohort	0–5 days before delivery	249	Fundal	Fundal	Non-Cephalic Presentation
					Right Cornual Fundal	High Lateral	
					Left Cornual Fundal	High Lateral	
					Anterior	Anterior	
					Posterior	Posterior	
					Previa	Previa	
Fried [14]	1978	Prospective Cohort	10–40 weeks	800	Anterior	Anterior	Non-Cephalic Presentation
					Posterior	Posterior	
					Anterior-to-Fundal	Central/Fundal	
					Posterior-to-Fundal	Central/Fundal	
					Fundal	Fundal	
Fung [15]	2011	Retrospective Cohort	14–23	16,236	Central	Central	Non-Cephalic Presentation
					Fundal	Fundal	Non-Elective Cesarean Section
					Lateral	Lateral	PPH
					Covering Os	Previa	Retained Placenta
							FGR
							Macrosomia
							APGAR < 7
							NICU Admission
							IUFD
							Neonatal Death
Gizzo [16]	2015	Prospective Cohort		1056	Fundal	Fundal	Non-Cephalic Presentation
					Lateral	Lateral	Non-Elective Cesarean Section
					Anterior	Anterior	
					Posterior	Posterior	
Granfors [17]	2020	Retrospective Cohort	16–22	3921	Fundal	Fundal	Retained Placenta
					Lateral	Lateral	
					Anterior	Anterior	
					Posterior	Posterior	
Granfors [18]	2019	Retrospective Cohort	18–22	74,087	Fundal	Fundal	Non-Cephalic Presentation
					Lateral	Lateral	PPH
					Anterior	Anterior	Retained Placenta
					Posterior	Posterior	SGA LGA APGAR < 7
Hill [19]	1982	Prospective Cohort	Mid-trimester	562	Anterior	Anterior	SAB/IUFD
					Posterior	Posterior	
					Fundal	Fundal	
					Lateral	Lateral	
					Previa	Previa	
Ito [20]	1998	Prospective Cohort	33–38 weeks	106	Central	Central	SGA
					Lateral	Lateral	
Jarvinen [21]	1966	Prospective Cohort	“End of Pregnancy”	279	Miduterine-Anterior	Anterior	Non-Cephalic Presentation
					Miduterine-Posterior	Posterior	
					High-Anterior	Fundal	
					High-Posterior	Fundal	
					Low-Anterior	Low	
					Low-Posterior	Low	
Kalanithi [22]	2007	Retrospective Case Control	16–20 week	272	Anterior	Anterior	SGA
					Posterior	Posterior	
					Lateral	Lateral	
					Fundal	Fundal	
					Low Lying	Low	
					Previa	Previa	
Kian [23]	1963	Prospective Cohort	Immediately after birth of child	362	Mid-Anterior	Anterior	Non-Cephalic Presentation
					Mid-Posterior	Posterior	
					Mid-Right Lateral	Lateral	
					Mid-Left Lateral	Lateral	
					Central Fundal	High Fundal	
					Anterior-Fundal	Central/Fundal	
					Posterior-Fundal	Central/Fundal	
					Right Cornual Fundal	High Lateral	
					Left Cornual Fundal	High Lateral	
					Low	Low	
Kofinas [24]	1989	Prospective Cohort	3rd Trimester or 24–28 weeks	300	Central	Central	SGA
					Unilateral	Lateral	
Li [25]	2019	Prospective Cohort	20–25	18	Previa	Previa	PPH
Liberati [26]	1997	Retrospective Cohort	22–24	732	Central	Central	SGA
					Lateral	Lateral	
Little [27]	1964	Retrospective Cohort	Exam of Placenta after Delivery	621	0	Low	APGAR < 7
					1–5	High (Above LUS)	
					6–10	High (Above LUS)	
					>10 cm	High (Above LUS)	
Lurie [28]	2003	Retrospective Cohort	3rd Trimester	200	Fundal	Central/Fundal	Retained Placenta
					Lateral	Central/Lateral	
					Anterior	Central/Anterior	
					Fundal	Central/Fundal	
Magann [29]	2007	Prospective Cohort	14–22	3336	Fundal High	Fundal	Non-Cephalic Presentation
					Lateral High	Lateral	PPH
					Low	Central/Low	FGR
							Macrosomia
							APGAR < 7
							IUFD
Meyer [30]	2020	Retrospective Cohort	Unknown	16,867	Fundal	Fundal	Retained Placenta
					Lateral	Lateral	
					Anterior/Posterior	Central	
Ogueh [31]	2003	Prospective Cohort	16–22	7641	Normal (“Upper Uterine Segment”)	Central/Segment	Non-Elective Cesarean Section
					Low Lying	Central/Lying	PPH
							SGA
Orgul [32]	2021	Retrospective Cohort	Unknown	69	Low Lying	Low	PPH
					Previa	Previa	
Osmundson [33]	2013	Retrospective Cohort	18–23	763	Low Lying	Low	PPH
					Previa	Previa	
					Normal	High (Above LUS)	
Porto [34]	2020	Retrospective Cohort	Mid-trimester	1203	Central	Central	Non-Elective Cesarean Section
					Lateral	Lateral	SGA
							APGAR < 7
							NICU Admission
Robinson [35]	2012	Prospective Cohort	Mid-trimester	1662	>30 mm (from os)	High (Above LUS)	Non-Elective Cesarean Section
					</=30 mm	Low	PPH
							SGA
							NICU Admission
							Neonatal Death
Salama-Bello [36]	2019	Retrospective Cohort	18–36 weeks	464	Central	Central	SGA
					Lateral	Lateral	
Seckin [37]	2015	Retrospective Cohort	18–24	1052	Central	Central	Non-Elective Cesarean Section
					Lateral	Lateral	PPH
							SGA
							Macrosomia
							APGAR < 7
							NICU Admission
Sekulic [38]	2013	Prospective Cohort	≥37	2750	Cornual-Fundal	High Lateral	Non-Cephalic Presentation
					Non-Cornual-Fundal	Midline	
Shah [39]	2021	Prospective Cohort	18–24	397	Central	Central	PPH
					Lateral	Lateral	FGR
							APGAR < 7
							NICU Admission
Talas [40]	2008	Prospective Case Control	≥36	350	Cornu-Fundus	High Lateral	Non-Cephalic Presentation
					Anterior Wall	Anterior	
					Posterior Wall	Posterior	
					Right Side of Wall	Lateral	
					Left Side of Wall	Lateral	
Torricelli [41]	2014	Prospective Cohort	Upon admission for delivery	2354	Anterior	Anterior	PPH
					Posterior	Posterior	Retained Placenta
					Fundal	Fundal	
Whitehead [42]	1953	Prospective Cohort	≥34	500	Anterior	Anterior	Non-Cephalic Presentation
					Posterior	Posterior	
					Antero-Lateral	3/4 Lateral	
					Lateral	Lateral	
					Fundal	Fundal	
					Cornual	High Lateral	
					Anterior Previa	Previa	
					Posterior Previa	Previa	
Zia [43]	2013	Retrospective Cohort	20–38 weeks	474	Anterior	Anterior	SGA
					Posterior	Posterior	IUFD
					Fundal	Fundal	
Legend:							
PPH	Post-Partum Hemorrhage						
SGA	Small for Gestational Age						
FGR	Fetal Growth Restriction						
LGA	Large for Gestational Age						
NICU	Neonatal Intensive Care Unit						
SAB	Spontaneous Abortion						
IUFD	Intrauterine Fetal Demise						

## Supplementary Materials

The following supporting information can be downloaded at: https://www.mdpi.com/article/10.3390/jcm14051649/s1, Table S1: MOOSE (Meta-analyses Of Observational Studies in Epidemiology) Checklist; Table S2: Newcastle-Otawa Scale (NOS); Fetal/Neonatal Complications: APGAR < 7 at 5 Minutes, NICU Admission, IUFD, Neonatal Death.
