# Supplementary material for: Placental Location Site and Associated Intrapartum, Postpartum, and Neonatal Complications: A Comprehensive Review and Meta-Analysis
Source: J Clin Med. 2025 Feb 28;14(5):1649. doi: 10.3390/jcm14051649 (PMC11899809; doi:10.3390/jcm14051649)
Supplement: Supplementary file 1 [file jcm-14-01649-s001.zip › jcm-3469417-Supplementary Materials.pdf]

A reporting checklist for Authors, Editors, and Reviewers of Meta-analyses of Observational Studies. You must report the page number in your manuscript where you consider each of the items listed in this checklist. If you have not included this information, either revise your manuscript accordingly before submitting or note N/A.

Table S1. MOOSE (Meta-analyses Of ObserYational Studies in Epidemiology) Checklist

Reporting Criteria	Reported (Yes/No)	Reported on Page No.
**Reporting of Background**		
Problem definition	Yes	3
Hypothesis statement	Yes	3-4
Description of Study Outcome(s)	Yes	3
Type of exposure or intervention used	Yes	4
Type of study design used	Yes	4
Study population	Yes	4
**Reporting of Search Strategy**		
Qualifications of searchers (eg, librarians and investigators)	Yes	4
Search strategy, including time period included in the synthesis and keywords	Yes	4
Effort to include all available studies, including contact with authors	Yes	4
Databases and registries searched	Yes	4
Search software used, name and version, including special features used (eg, explosion)	Yes	4
Use of hand searching (eg, reference lists of obtained articles)	Yes	4
List of citations located and those excluded, including justification	Yes	4-5 and Fig. 1
Method for addressing articles published in languages other than English	Yes	4-5
Method of handling abstracts and unpublished studies	Yes	5
Description of any contact with authors	No	
**Reporting of Methods**		
Description of relevance or appropriateness of studies assembled for assessing the hypothesis to be tested	Yes	5
Rationale for the selection and coding of data (eg, sound clinical principles or convenience)	Yes	6
Documentation of how data were classified and coded (eg, multiple raters, blinding, and interrater reliability)	Yes	6
Assessment of confounding (eg, comparability of cases and controls in studies where appropriate	Yes	6-12

Reporting Criteria	Reported (Yes/No)	Reported on Page No.
Assessment of study quality, including blinding of quality assessors; stratification or regression on possible predictors of study results	Yes	6-12
Assessment of heterogeneity	Yes	6-12
Description of statistical methods (eg, complete description of fixed or random effects models, justification of whether the chosen models account for predictors of study results, dose-response models, or cumulative meta-analysis) in sufficient detail to be replicated	Yes	6-12
Provision of appropriate tables and graphics	Yes	6-12
**Reporting of Results**		
Table giving descriptive information for each study included	Yes	NOS Scale App
Results of sensitivity testing (eg, subgroup analysis)	Yes	6-12
Indication of statistical uncertainty of findings	Yes	6-12
**Reporting of Discussion**		
Quantitative assessment of bias (eg, publication bias)	Yes	12-13
Justification for exclusion (eg, exclusion of non–English-language citations)	Yes	12-13
Assessment of quality of included studies	Yes	12-13
**Reporting of Conclusions**		
Consideration of alternative explanations for observed results	Yes	12-14
Generalization of the conclusions (ie, appropriate for the data presented and within the domain of the literature review)	Yes	13-14
Guidelines for future research	Yes	14-15
Disclosure of funding source	Yes	1

Once you have completed this checklist, please save a copy and upload it as part of your submission. DO NOT include this checklist as part of the main manuscript document. It must be uploaded as a separate file.

Table S2. Newcastle-Ottawa Scale (NOS)

	Author	Year Published	Study Design	NOS Score Risk of Bias		
				Selection	Comparability	Outcome/Exposure
1	Baba	2014	Retrospective Case Control	***	*	***
2	Bardin	2022	Retrospective Cohort	****	**	***
3	Becker	2001	Prospective Cohort	****	*	**
4	Belachew	2017	Prospective Cohort	****	**	***
5	Chapman	1979	Prospective Cohort	****	*	**
6	Chen	2020	Retrospective Cohort	****	*	***
7	Contro	2010	Prospective Cohort	****	**	***
8	Davydov	1987	Retrospective Cohort	*	-	**
9	Devarajan	2012	Retrospective Cohort	***	*	***
10	Fianu	1978	Prospective Cohort	***	*	**
11	Fried	1978	Prospective Cohort	**	*	*
12	Fung	2011	Retrospective Cohort	****	**	***
13	Gizzo	2015	Prospective Cohort	***	*	**
14	Granfors	2019	Retrospective Cohort	****	*	**
15	Granfors	2020	Retrospective Cohort	****	*	***
16	Hill	1982	Prospective Cohort	***	*	**
17	Ito	1998	Prospective Cohort	**	*	**
18	Jarvinen	1966	Prospective Cohort	* *	*	*
19	Kalanithi	2007	Retrospective Case Control	****	*	***
20	Kian	1963	Prospective Cohort	**	-	*
21	Kofinas	1989	Prospective Cohort	***	*	**
22	Li	2019	Prospective Cohort	****	*	*
23	Liberati	1997	Retrospective Cohort	****	*	***
24	Little	1964	Retrospective Cohort	***	-	***
25	Lurie	2003	Retrospective Cohort	***	*	**
26	Magann	2007	Prospective Cohort	****	*	***
27	Meyer	2020	Retrospective Cohort	****	*	***
28	Ogueh	2003	Prospective Cohort	****	*	***
29	Orgul	2021	Retrospective Cohort	***	*	***
30	Osmundson	2013	Retrospective Cohort	****	**	**
31	Porto	2020	Retrospective Cohort	***	*	***
32	Robinson	2012	Prospective Cohort	****	**	***
33	Salama-Bello	2019	Retrospective Cohort	****	**	***
34	Seckin	2015	Retrospective Cohort	***	*	***

Newcastle-Ottawa Scale (NOS)

35	Sekulic	2013	Prospective Cohort	***	*	**
36	Shah	2021	Prospective Cohort	****	*	***
37	Talas	2008	Prospective Case Control	**	*	*
38	Torricelli	2014	Prospective Cohort	***	*	**
39	Whitehead	1953	Prospective Cohort	**	*	*
40	Zia	2013	Retrospective Cohort	****	*	***
